# Combined Transcriptomic and Metabolomic Analyses of the Response of *Ganoderma lucidum* to Elevated CO_2_

**DOI:** 10.3390/jof12010005

**Published:** 2025-12-20

**Authors:** Tingting Fang, Lu Chen, Hui Yao, Ye Li, Guohui Liu, Shaofeng Wu, Jin Lan, Xiangdong Chen

**Affiliations:** 1 Institute of Medicinal Plant Development, Chinese Academy of Medical Sciences & Peking Union Medical College, Beijing 100193, China; s2024009067@student.pumc.edu.cn (T.F.); s2025009074@student.pumc.edu.cn (L.C.); hyao@implad.ac.cn (H.Y.); 2 Fujian Xianzhilou Biological Science and Technology Co., Ltd., Fuzhou 300057, China; lee@xianzhilou.com (Y.L.); lgh36201@163.com (G.L.); sooshaofoong@163.com (S.W.)

**Keywords:** *Ganoderma lucidum*, carbon dioxide, transcriptomics, metabolomics, antler-shaped fruiting body

## Abstract

Carbon dioxide (CO_2_) is a key environmental factor that regulates the morphology of fruiting bodies in edible fungi. High CO_2_ concentrations often lead to the formation of antler-shaped abnormal fruiting bodies in *Ganoderma lucidum*. Yet, the molecular response mechanisms underlying this process remain unclear. To address this gap, this study integrated transcriptomics and untargeted metabolomics to compare the transcriptional and metabolic profiles of *G. lucidum* fruiting bodies at three growth stages, cultivated under both normal (0.04%) and high CO_2_ concentrations (0.3%). Metabolomic analysis revealed that, compared to the control groups, 387, 337, and 445 differentially accumulated metabolites were identified in the elevated-CO_2_ groups, respectively. Moreover, high CO_2_ concentrations led to a widespread down-regulation of various amino acids biosynthesis, accompanied by a marked accumulation of specific triterpenoids and steroids. This indicates distinct metabolite accumulation patterns in the fruiting bodies of *G. lucidum* cultivated under elevated CO_2_. Furthermore, transcriptomic analysis showed that, at a key stage of fruiting body development, high CO_2_ concentrations adversely affected gene expression of cell cycle-yeast, proteasome, DNA replication, mismatch repair, and meiosis-yeast pathways, which may decrease the cell division ability and prevent normal pileus development. Meanwhile, the differential expression of genes related to CO_2_ signal perception and transduction and cell wall remodeling provided a molecular basis for the morphogenesis of the antler-type fruiting bodies. Overall, this study delineates a multi-layered, multi-pathway regulatory network through which high CO_2_ concentrations affect the development and metabolism of *G. lucidum*, encompassing energy metabolism reprogramming, inhibition of cell division, and cell wall remodeling. This provides new insights into CO_2_ as an environmental signal in fungal development and a theoretical basis for optimizing *G. lucidum* cultivation practices.

## 1. Introduction

*Ganoderma lucidum*, known as lingzhi in China, boasts a 4000-year history of economic and medicinal significance [1]. Modern studies have shown that *G. lucidum* contains a variety of biologically active components, primarily polysaccharides, triterpenoids, nucleotides, steroids, peptides, unsaturated fatty acids, and amino acids [2]. Among these, triterpenoids and polysaccharides are the main bioactive compounds in *G. lucidum*, possessing a wide range of pharmacological properties, including anti-inflammatory [3], anti-tumor [4], anti-fatigue [5], antioxidant [6], hypoglycemic [7], cardiovascular disease prevention [8], hepatoprotective [9], and neuroprotective effects [10]. With an increasing understanding of the efficacy of *G. lucidum*, market demand continues to rise, highlighting the urgency of developing high-quality and efficient cultivation practices of *G. lucidum.*

Numerous studies have shown that CO_2_ concentration levels influence the growth, development, morphology, and bioactive compound production in edible fungi. Tolerance to CO_2_ varies significantly among different species, developmental stages, and even different tissues of the fungi. In recent years, a preliminary understanding of the effects of CO_2_ concentration on commercially cultivated species, such as *Agaricus bisporus* [11], *Flammulina velutipes* [12], *Pleurotus ostreatus* [13], and *Schizophyllum commune* [14,15], has emerged, and this knowledge now guides practical cultivation practices. In many mushroom species, high CO_2_ concentrations inhibit pileus development and expansion, while promoting stipe elongation and thinning. Although the impact of CO_2_ concentration on the growth and development of fruiting bodies in edible fungi has been well-documented, the underlying regulatory mechanisms, particularly at the molecular level, remain unclear. Previous studies demonstrated that CO_2_ can modulate morphological development, mating, sporulation, phenotypic switching, and virulence processes of fungi via the adenylyl cyclase/cAMP pathway [16]. Furthermore, Lu et al. found that CO_2_ signaling suppresses the phosphorylation and degradation of Ume6, thereby regulating fungal hyphal development [17]. Recent studies employing transcriptomics and proteomics have explored the molecular mechanisms underlying the impact of CO_2_ on edible mushroom fruiting body development, suggesting that elevated CO_2_ may disrupt gene expression and protein function associated with the cell cycle, cell wall biosynthesis, as well as energy metabolism [12,18,19]. While these omics studies have begun to elucidate the molecular responses of various edible fungi to CO_2_ stress, the specific mechanisms in *G. lucidum*, particularly concerning its prized bioactive compounds and unique morphological adaptations, remain largely unexplored.

Research indicates that *G. lucidum* is an aerobic fungus. When the CO_2_ content in the air increases to 0.1%, it promotes stipe growth while inhibiting pileus elongation. When the CO_2_ content reaches 0.1~1%, it is easy to form antler-shaped deformed fruiting bodies. Antler-shaped *G. lucidum* has been reported to contain more triterpenoids, carbohydrates, and fiber, exhibiting stronger immunostimulatory activity than that of common kidney-shaped *G. lucidum* [20,21,22]. Surya et al. cultivated antler-shaped *G. lucidum* by manipulating the conditions of ventilation and light, and found that the concentration of CO_2_ gas up to 2–5% elicited the bioactive compounds in this antler mushroom, resulting in higher antioxidant potential [23]. This suggests that elevated CO_2_ may have a significant impact on the morphological development and physiological metabolism of *G. lucidum*, providing new ideas for its high-quality and efficient cultivation.

However, current research on the molecular mechanisms underlying the growth and development of *G. lucidum* under elevated CO_2_ conditions is relatively limited, particularly the mechanisms by which elevated CO_2_ affects gene expression and metabolic pathways in *G. lucidum* remain unclear. Transcriptomics and metabolomics, respectively, analyze the physiological state and functional changes in organisms at the levels of gene expression and metabolites, playing a key role in deciphering fungal metabolic pathways and the biosynthesis of bioactive compounds [24,25]. In this study, transcriptomics and metabolomics analyses were employed to investigate the effects of high CO_2_ concentration on the morphogenesis and secondary metabolism of *G. lucidum* fruiting bodies. We seek to elucidate the formation mechanism of the antler-shaped fruiting bodies, thereby providing a theoretical basis for the high-quality and high-yield cultivation of *G. lucidum* and the precise regulation of its bioactive compounds.

## 2. Materials and Methods

### 2.1. Culture Conditions and Acquisition of the G. lucidum Samples

The tested *G. lucidum* samples were collected from a cultivation base in Pucheng County, Nanping City, Fujian Province (28°0′53.64″ N, 118°31′26.112″ E). These samples were taxonomically identified as *Ganoderma lucidum (Leyss. ex Fr.) Karst.* by Prof. Lan Jin from the Institute of Medicinal Plant Development, Chinese Academy of Medical Sciences. The experimental strains were initially cultured on potato dextrose agar (PDA) medium (200 g potatoes, 20 g glucose, 20 g agar, 1 L deionized water) at 25 °C for 7 days. After the mycelia fully colonized the medium, the culture was used for bag inoculation.

The cultivation substrate consisted of sawdust-bran mixture (84% sawdust, 15% wheat bran, 1% gypsum powder, 60% moisture content). Each polypropylene plastic bag (15 cm × 30 cm) was filled with 800 g of cultivation substrate, sterilized at 121 °C for 2 h, and then inoculated with a quantified amount of spawn. The bags were incubated at 25 °C under dark conditions, and the entire substrate was fully colonized by the mycelium after 25 days. Next, bags were transferred to the mushroom-growing room for fruiting body induction. The CO_2_ concentration in the room was monitored with a CO_2_ controller and maintained at 0.04% (normal CO_2_) or 0.3% (elevated CO_2_) by injecting CO_2_ or O_2_ from gas cylinders. Both the control group and elevated-CO_2_ group were incubated in an atmosphere with 80% humidity, 12 h light/12 h dark, and at 25 °C. To investigate the metabolic regulation by elevated CO_2_ during post-fruiting developmental phases of *G. lucidum*, three time points (10 d, 20 d, and 43 d) were selected as harvest times. Fruiting body samples were collected at these time points from both the control group (designated as J, K, L) and the CO_2_-enriched treatment group (designated as M, N, O). Each group included three biological replicates. Harvested samples were immediately frozen in liquid nitrogen and stored at −80 °C for subsequent metabolomic and transcriptomic analyses.

### 2.2. Metabolomic Analysis

#### 2.2.1. Sample Preparation and LC-MS Analysis

After the harvested fruiting body samples were ground with liquid nitrogen, 25 mg of the powdered sample was weighted to a microcentrifuge tube and 500 μL extract solution (methanol/water = 3:1, with isotopically-labeled internal standard mixture) was added. Next, the samples were processed for 4 min at 35 Hz in a grinder (MM 400, Retsch, Haan, Germany) and sonicated for 5 min in an ice-water bath. The homogenization and sonication cycle was repeated 3 times. Then the samples were incubated for 1 h at −40 °C and centrifuged at 12,000 rpm (RCF = 13,800× *g*, R = 8.6 cm) for 15 min at 4 °C. The resulting supernatant was collected, filtered through a 0.22 µm microporous membrane, transferred to an LC injection vial, and stored at −80 °C until LC-MS analysis. The quality control (QC) sample was prepared by mixing the extracts of all samples in equal volume, which was used to judge the stability of experiments and instruments in the process of sample detection. All the measurements were carried out in triplicate.

LC-MS/MS analyses were carried out on a UHPLC system (Vanquish, Thermo Fisher Scientific, Mumbai, India) linked to a Q Exactive HFX mass spectrometer (Orbitrap MS, Thermo, Waltham, MA, USA) and fitted with an ACQUITY UPLC HSS T3 column (Waters Corporation, Bengaluru, India) (2.1 mm × 100 mm, 1.8 μm). For the mobile phase, solvent A was an aqueous solution of 25 mmol/L ammonium acetate and 25 mmol/L ammonium hydroxide, while solvent B was acetonitrile. The auto-sampler temperature was held at 4 °C, and 3 μL was injected for each analysis.

The primary and secondary mass spectra were acquired using the QE HFX mass spectrometer (Thermo Fisher Scientific, Waltham, MA, USA) in information-dependent acquisition (IDA) mode in the control of the acquisition software (Xcalibur 4.5, Thermo, West Etobicoke, ON, Canada). In this mode, the acquisition software continuously evaluates the full scan MS spectrum. The following instrument settings were used: ion source: ESI; sheath gas flow rate: 30 Arb; aux gas flow rate: 25 Arb; capillary temperature: 350 °C; full MS resolution: 60,000; MS/MS resolution: 7500; collision energy: 10/30/60 in NCE mode; spray Voltage: 3.6 kV in positive ion mode and −3.2 kV in negative ion mode.

#### 2.2.2. Metabolite Identification and Quantification

Metabolite identification was achieved by matching experimental MS/MS spectra against an in-house self-built secondary mass spectral database to obtain accurate qualitative and relative quantitative results. The cutoff for annotation was set at 0.3. In addition, metabolite features detected in less than 20% of the experimental samples or in less than 50% of the pooled QC samples were removed, and the remaining missing values were substituted with half of the minimum value. Features with a relative standard deviation (RSD) greater than 30% were removed to ensure the stability and reproducibility of the measurements before downstream statistical analysis.

The compounds were identified by searching in the KEGG database (https://www.kegg.jp/kegg/kegg1.html, accessed on 7 October 2025), HMDB database (https://hmdb.ca/, accessed on 7 October 2025), METLIN database (https://metlin.scripps.edu, accessed on 7 October 2025), PubChem database (https://pubchem.ncbi.nlm.nih.gov/, accessed on 7 October 2025) and ChEBI database (http://www.ebi.ac.uk/chebi/, accessed on 7 October 2025). Principal Component Analysis (PCA) and Orthogonal Partial Least Squares Discriminant Analysis (OPLS-DA) were conducted in R to analyze the overall metabolic differences, the variability between groups, and the diversity between samples. Based on the OPLS-DA model, the Variable Importance in Projection (VIP) score was used to preliminarily screen differential metabolites. The *p*-values were obtained from a double-tailed Student’s *t*-test on normalized peak areas. Metabolites with VIP ≥ 1 and *p*-value < 0.05 were selected as differentially accumulated metabolites (DAM).

### 2.3. Transcriptome Analysis

#### 2.3.1. Transcriptome Sequencing

The extraction of RNA, the construction of cDNA Library, and the RNA sequencing (RNA-seq) were performed. According to the manufacturer’s instructions, total RNA was isolated from freeze-dried fruiting bodies of *G. lucidum* using the TriZol reagent (Life Technologies, Carlsbad, CA, USA). The quality of the extracted RNA was confirmed by measuring its concentration and purity on a NanoDrop 2000 (Thermo Fisher Scientific, Wilmington, DE, USA) and evaluating its integrity using the RNA Nano 6000 Assay Kit with an Agilent 2100 Bioanalyzer (Agilent Technologies, Santa Clara, CA, USA). Sequencing libraries were built using Hieff NGS Ultima Dual-mode mRNA Library Prep Kit for Illumina (Yeasen Biotechnology (Shanghai) Co., Ltd., Shanghai, China) and index codes were added to attribute sequences to each sample. The 250~300 bp cDNA fragments were screened with the AMPure XP system (Beckman Coulter Life Sciences, Brea, CA, USA) and then amplified by PCR to obtain the final library. Following quality assessment of the PCR products on the Agilent Bioanalyzer 2100, the libraries were sequenced on the Illumina NovaSeq 6000 to yield 150 bp paired-end reads.

#### 2.3.2. Transcriptome Data Analysis

Raw reads were further processed on the BMKCloud (www.biocloud.net) online platform. Initial quality control was performed with in-house perl scripts to filter raw FASTQ files. This process involved removing reads containing adapters, poly-N sequences, or low-quality bases to generate high-quality clean data. The resulting clean reads were mapped to the reference genome of *G. lucidum* G.260125-1 (Project accession number PRJNA71455) using Hisat2 v2.2.1 software. Next, the StringTie Reference Annotation Based Transcript (RABT) assembly method was employed to construct and identify both known and novel transcripts from Hisat2 alignment results. Subsequently, the resulting transcripts were compared against multiple public databases, including Nr (NCBI non-redundant protein sequences), Pfam (Protein family), KOG/COG (Clusters of Orthologous Groups of proteins), Swiss-Prot (A manually annotated and reviewed protein sequence database); KO (KEGG Ortholog database); GO (Gene Ontology), to obtain functional annotation information for the transcripts.

The levels of gene expression were estimated using FPKM (fragments per kilobase of transcript per million fragments mapped). To identify differentially expressed genes (DEGs) between sample groups, the R package DESeq2 v1.30.1 was used for differential expression analysis. The resulting *p*-values were adjusted using the Benjamini and Hochberg’s approach for controlling the false discovery rate. Genes with FDR < 0.01 and |Fold Change| ≥ 2 were assigned as significantly differentially expressed. Lastly, GO (Gene Ontology) and KEGG (Kyoto Encyclopedia of Genes and Genomes) enrichment analyses of DEGs were implemented by clusterProfiler v4.4.4 to determine the main affected biological functions or pathways.

## 3. Results

### 3.1. Comparison of Metabolomic Data

#### 3.1.1. Quality Control of the Metabolome Data

**Figure 1:**
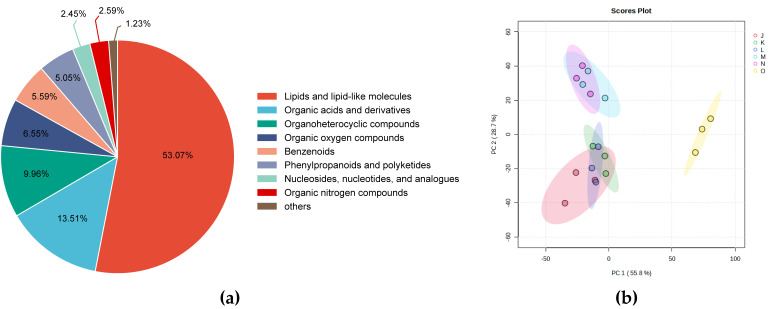
Quality Control of the Metabolome Data. (**a**) Pie chart of the percentage distribution of different metabolite classes; (**b**) PCA of metabolomics data.

Non-targeted metabolomics was employed to analyze the metabolic profiles of *G. lucidum* across three growth stages in both control and high CO_2_ groups. A total of 745 metabolites were identified in the samples, of which 733 were classified into 9 groups: 389 lipids and lipid-like molecules, 99 organic acids and derivatives, 73 organoheterocyclic compounds, 48 organic oxygen compounds, 41 benzenoids, 37 phenylpropanoids and polyketides, 18 nucleosides, nucleotides and analogs, 19 organic nitrogen compounds, and 9 other components (Figure 1a and Supplementary Table S1). In the principal component analysis (PCA) diagram, *G. lucidum* samples were separated into distinct clusters based on different developmental stages of the control group and the treatment group (Figure 1b). The first principal component (PC1) explained 55.8% of the total variance and the second principal component (PC2) separated samples with a 28.7% variance contribution value, effectively illustrating the differences among the samples. Among them, the samples in the control group (J, K, L) were clearly distinguished from those in the high-concentration CO_2_ group (M, N, O), indicating the significant differences in metabolite levels among *G. lucidum* fruiting bodies grown under different CO_2_ conditions. Notably, fruiting bodies in the third growth stage (O) of the high-concentration CO_2_ group were clearly separated from other samples, suggesting that the overall metabolism of *G. lucidum* exposed to high concentrations of CO_2_ for a long time underwent significant changes in the later stages of growth and development.

#### 3.1.2. Differential Metabolic Profile of *G. lucidum* Cultivated Under Elevated CO_2_ Conditions

Pairwise comparative analysis was conducted between control and CO_2_-elevated treatment groups across three developmental stages to identify differences in the metabolite accumulation patterns.

**Figure 2:**
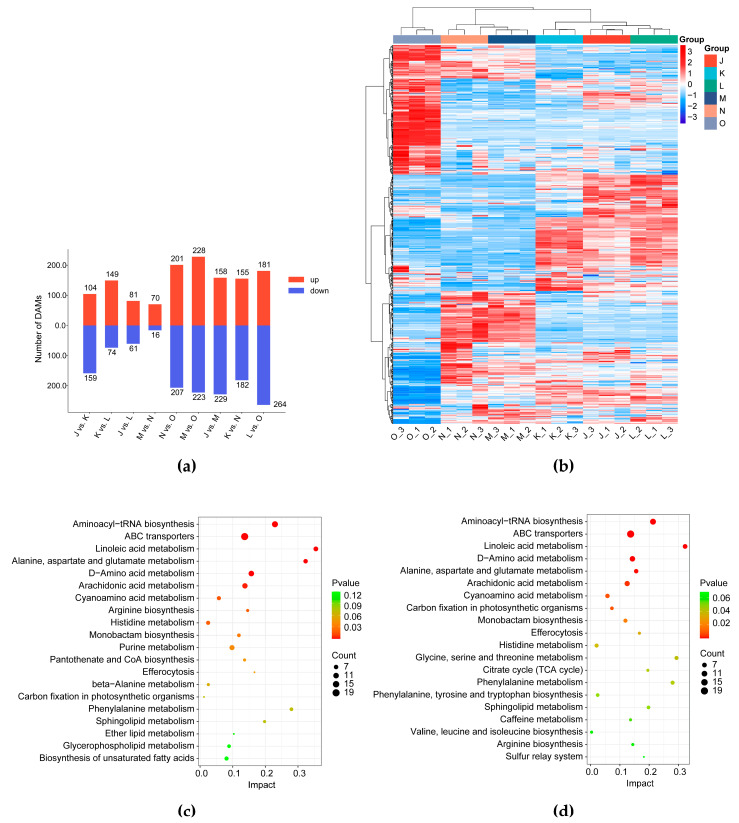

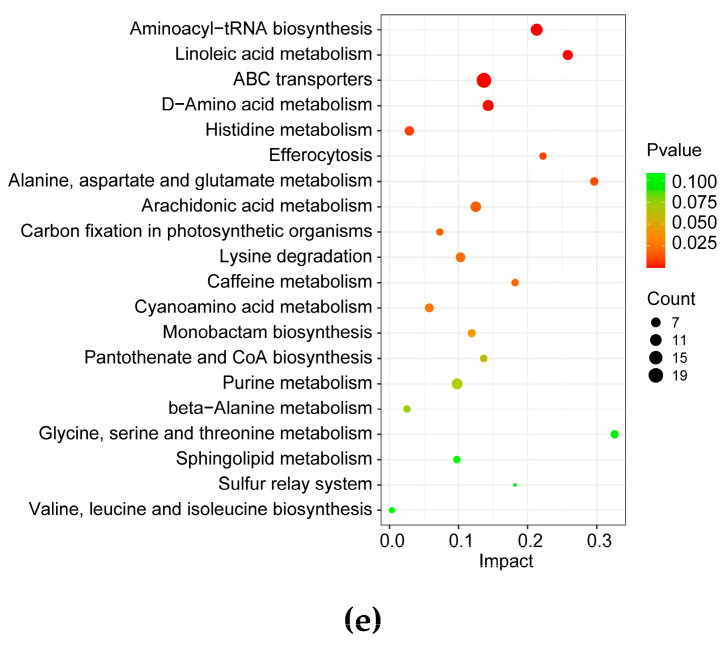
Metabolic characteristics of *G. lucidum* cultivated under different CO_2_ conditions. (**a**) Statistical analysis of DAMs. (**b**) The heatmap of metabolic data. Each column and row represent a sample and a DAM, respectively. The color gradient indicates the normalized relative abundance, with red representing high abundance and blue representing low abundance. (**c**–**e**) KEGG enrichment analysis of the DAMs in J vs. M (**c**), K vs. N (**d**), and L vs. O (**e**). The *x*-axis represents the richness factor and the *y*-axis indicates the KEGG pathways. The size of the bubble represents the number of DAMs in the pathway, and the color corresponds to the *p*-value (redder colors indicate higher significance).

The differentially accumulated metabolites (DAMs) were screened based on VIP ≥ 1 and *p* < 0.05. This analysis yielded 625 DAMs at three developmental stages of both control and CO_2_-enriched treatment groups (nine pairwise comparison sets: J vs. K; K vs. L; J vs. L; M vs. N; N vs. O; M vs. O; J vs. M; K vs. N; L vs. O) (Supplementary Table S2, Figure S1). Specifically, intra-group temporal comparisons within the control group (J vs. K; K vs. L; J vs. L) identified 263, 223, and 142 DAMs, respectively (Figure 2a). Conversely, the CO_2_-treated group exhibited distinct dynamics, with M vs. N, N vs. O, and M vs. O comparisons yielding 86, 408, and 451 DAMs correspondingly. Meanwhile, cross-group analysis at equivalent developmental phases demonstrated CO_2_-induced metabolic perturbations: 387, 337 and 445 DAMs were identified in J vs. M, K vs. N, and L vs. O, respectively. These findings were further confirmed by Venn diagrams and the heatmap of hierarchical clustering of DAMs (Figure 2b).

To identify the affected metabolic pathways, we mapped the DAMs identified in the J vs. M, K vs. N, and L vs. O comparisons onto the KEGG pathway database. Among the enriched pathways, aminoacyl-tRNA biosynthesis, ABC transporters, linoleic acid metabolism, alanine, aspartate and glutamate metabolism, D-Amino acid metabolism, arachidonic acid metabolism, cyanoamino acid metabolism, histidine metabolism, monobactam biosynthesis, efferocytosis were consistently identified across all comparisons, underscoring their crucial functions in key cellular processes such as protein synthesis, nitrogen metabolism, and intracellular transport (Figure 2c–e). These pathways are vital for the growth and adaptive metabolism of *G. lucidum* under high CO_2_ stress, as amino acid biosynthesis directly provides precursors for protein synthesis, supporting cell proliferation and repair, while ABC transporters accelerate nutrient uptake and toxic substance efflux to maintain intracellular homeostasis under CO_2_ stress. Key DAMs in these pathways include various amino acids (L-Alanine, L-Aspartic acid, L-Arginine, L-Serine, L-Tyrosine, L-Histidine, L-Proline, L-Asparagine, L-Valine, L-Threonine, 5-Aminopentanoic acid, D-Glutamine, 1-Methylhistidine) and carbohydrates (Sucrose, Mannitol, Sorbitol). These findings indicate that the changes in the levels of amino acids and their derivatives, energy metabolites, and carbohydrates may provide a potential metabolic basis for the alterations in morphogenesis in *G. lucidum* cultivated under high CO_2_ conditions.

#### 3.1.3. Effects of Elevated CO_2_ Conditions on Bioactive Compounds in the *G. lucidum* Fruiting Body

**Figure 3:**
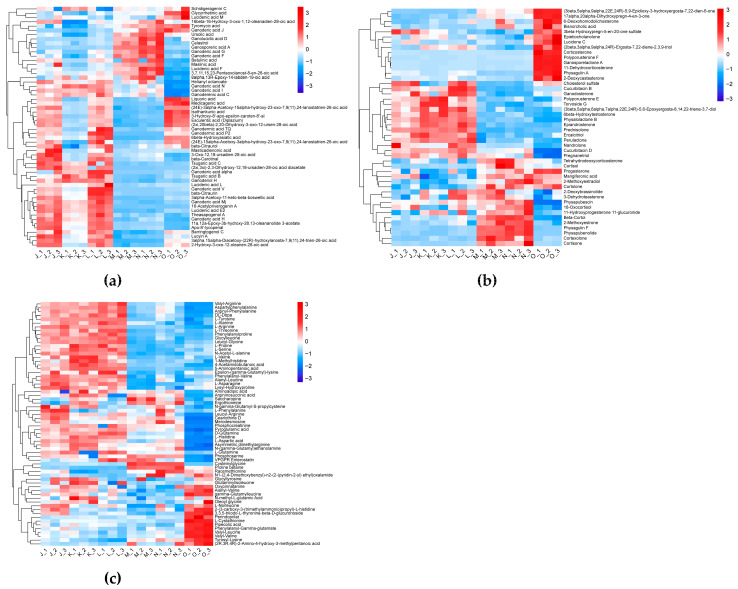
Differential landscape of bioactive compounds in *G. lucidum* cultivated under both normal and high CO_2_ concentrations; (**a**) Heatmap of 56 differentially accumulated triterpenoids; (**b**) Heatmap of 46 differentially accumulated steroids and steroid derivatives; (**c**) Heatmap of 64 differentially accumulated amino acids, peptides, and analogs.

Triterpenoids are the main active components of *G. lucidum* and serve as a key indicator for its quality evaluation. Among the 64 triterpenoids identified, 56 exhibited differential accumulation in at least one comparison (Figure 3a). In the J vs. M group, 46 triterpenoids were differentially accumulated, with 37 decreasing and only 9 increasing. In the K vs. N group, 45 triterpenoids were differentially accumulated, with 29 decreasing and 16 increasing. In the L vs. O group, 11 triterpenoids increased while 30 decreased.

Steroids, an important class of secondary metabolites in *G. lucidum*, have various medical applications. Metabolomic analysis revealed that elevated CO_2_ cultivation conditions significantly affect the accumulation of steroid compounds in the *G. lucidum* fruiting body. In the J vs. M, K vs. N, and L vs. O comparisons, 33, 28, and 41 differentially accumulated steroids were detected, respectively (Figure 3b).

*G. lucidum* is rich in amino acids, including all 8 essential and 10 non-essential types. This study found that under elevated CO_2_ cultivation conditions, amino acids, peptides, and analogs in the *G. lucidum* fruiting body showed differential accumulation (Figure 3c). Specifically, in J vs. M, 35 of these DAMs were down-regulated and 8 were up-regulated. In K vs. N, 32 were down-regulated and 5 were up-regulated. In L vs. O, 36 were down-regulated and 17 were up-regulated. These results revealed that high CO_2_ concentrations adversely affect the synthesis and accumulation of amino acids and peptides in the fruiting bodies of *G. lucidum*.

### 3.2. Comparison of Transcriptomic Data

#### 3.2.1. Quality Assessment of Transcriptome Data

**Figure 4:**
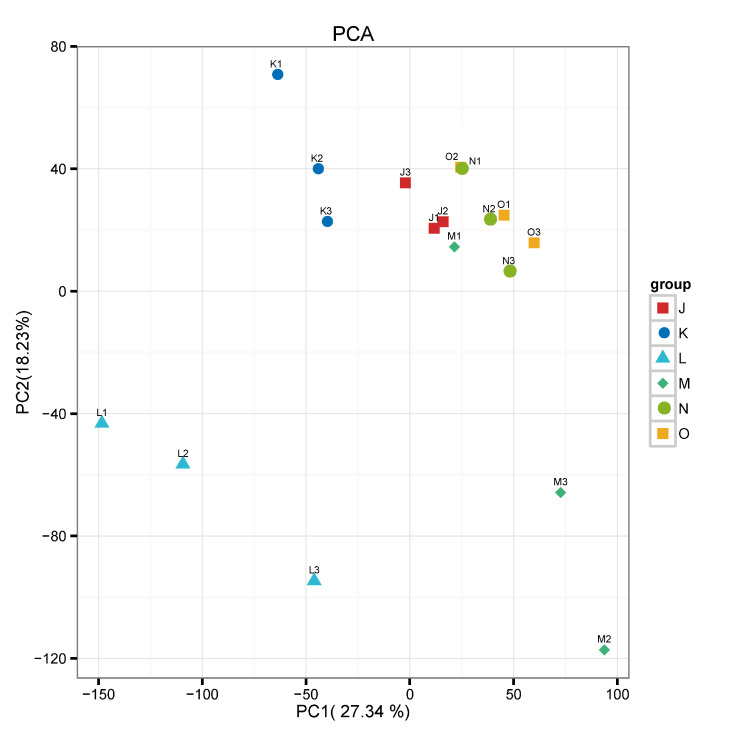
PCA of transcriptomic data.

To elucidate the molecular basis of gene regulation underlying the metabolic changes in *G. lucidum* under high CO_2_ concentration, the transcriptomes of 18 samples were sequenced. Following the removal of adapters and low-quality sequences, a total of 133.97 Gb of clean data were generated, characterized by Q20 ≥ 97.03% and Q30 ≥ 92.45% (Supplementary Table S3). Moreover, when clean reads were mapped to the reference genome, between 85.41 and 88.45% of clean reads were matched. To assess the biological repeatability, Pearson’s correlation analysis was conducted, displaying a high degree of correlation (R^2^ > 0.8) among all biological replicates (Figure S2). Meanwhile, principal component analysis revealed the good quality of the transcriptome data, with PC1 and PC2 accounting for 27.34% and 18.23% of the variation, respectively (Figure 4).

#### 3.2.2. Differential Transcriptomic Profile of *G. lucidum* Cultivated Under Elevated CO_2_ Conditions

**Figure 5:**
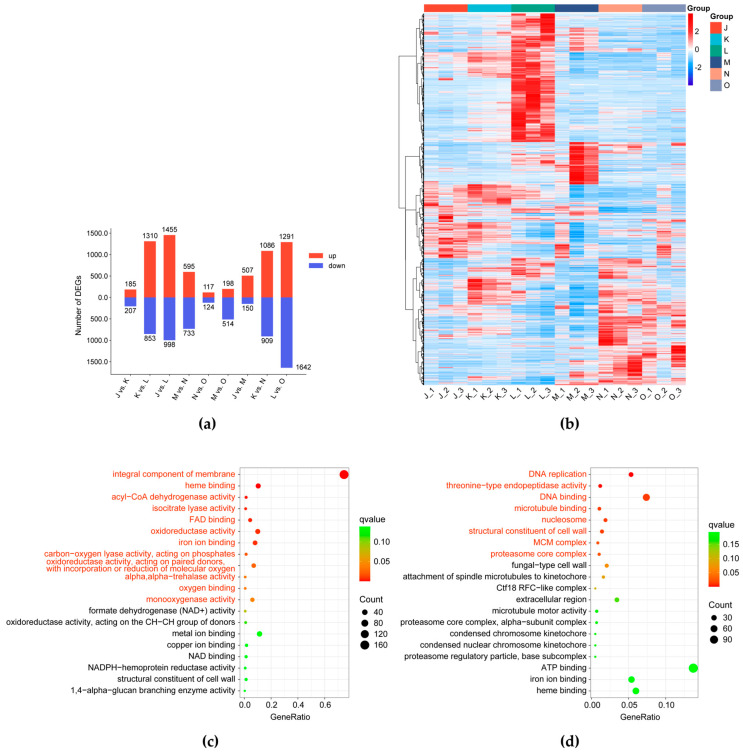

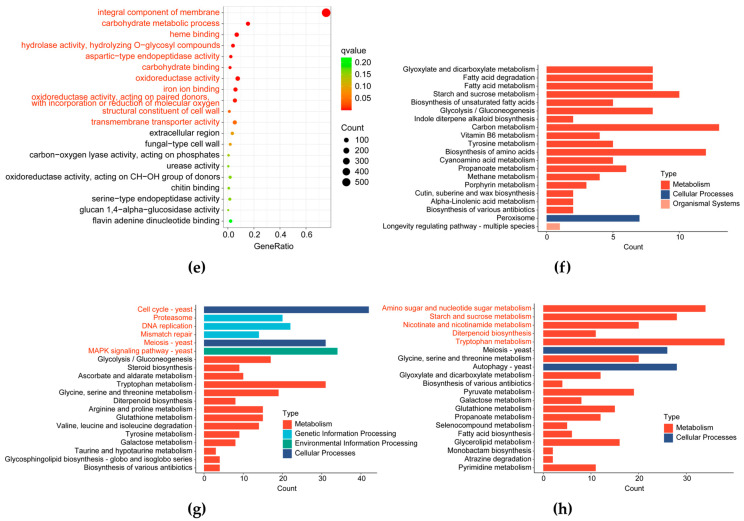
Transcriptomic characteristics of *G. lucidum* cultivated under different CO_2_ conditions. (**a**) Statistical analysis of DEGs.; (**b**) The heatmap of transcriptomic data. The rows represent individual DEGs and the columns represent the sample groups. The color gradient indicates the relative expression level, with red signifying high expression and blue signifying low expression; (**c**–**e**) GO enrichment bubble plot for DEGs in J vs. M (**c**), K vs. N (**d**), and L vs. O (**e**). The *x*-axis shows the ratio of the number of DEGs to the total number of genes annotated within a specific GO term (representing enrichment level) and the *y*-axis represents the GO term, respectively. The size of the bubble represents the number of DEGs in the term, and the color corresponds to the q-value (redder colors indicate higher significance). Terms highlighted in red text are significantly enriched (q < 0.05); (**f**–**h**) KEGG enrichment bar plot for the DEGs in J vs. M (**f**), K vs. N (**g**), and L vs. O (**h**). The *y*-axis lists the KEGG pathways and the number at the end of each bar represents the count of DEGs enriched in that pathway. Terms highlighted in red text are significantly enriched (q < 0.05).

Differentially expressed genes (DEGs) were identified from the nine comparative groups based on the criteria of a |Fold Change| ≥ 2 and an FDR < 0.01. Using FPKM data, a total of 5913 DEGs were identified among fruiting body samples (Figure 5a and Supplementary Table S4). Specifically, L vs. O contained the largest number of the DEGs (1291 up-regulated and 1642 down-regulated) and the DEGs in the J vs. M, K vs. N, and L vs. O groups increased sequentially, indicating that the number of DEGs between the control group and the high-CO_2_ cultivation group gradually increased as the fruiting bodies matured. The clustering heat map of DEGs further illustrated distinct gene expression patterns across the different groups in this experiment (Figure 5b).

GO and KEGG enrichment analyses were conducted to further investigate the functional roles of the DEGs. In the first growth stage, DEGs from the J vs. M comparison were significantly enriched in GO terms such as integral component of membrane, heme binding, acyl-CoA dehydrogenase activity, isocitrate lyase activity, FAD binding, oxidoreductase activity, and iron ion binding (Figure 5c). Notably, in the second growth stage, DEGs in the K vs. N comparison were primarily involved in DNA replication, DNA binding, nucleosome, and MCM complex, all of which are related to cell division and proliferation (Figure 5d). In the third growth stage, DEGs from the L vs. O comparison were significantly enriched in the integral component of the membrane, carbohydrate metabolic process, heme binding, hydrolase activity, hydrolyzing O-glycosyl compounds, aspartic-type endopeptidase activity, carbohydrate binding, and oxidoreductase activity (Figure 5e).

The KEGG enrichment analysis showed that the DEGs in J vs. M and L vs. O were mainly enriched in the metabolic pathways (Figure 5f,h). Notably, at the third growth stage of *G. lucidum* fruiting bodies under long-term high CO_2_ exposure, pathways such as amino sugar and nucleotide sugar metabolism, starch and sucrose metabolism, nicotinate and nicotinamide metabolism, diterpenoid biosynthesis, and tryptophan metabolism were significantly affected, which are closely related to the synthesis of bioactive compounds. Conversely, DEGs in the K vs. N comparison demonstrated significant enrichment in cell cycle-yeast, proteasome, DNA replication, mismatch repair, meiosis-yeast, and MAPK signaling pathway-yeast, with the majority of these genes being down-regulated (Figure 5g). This suggests that during this growth stage, high CO_2_ inhibits cell division and proliferation through these signaling pathways, thereby affecting primordia development and pileus expansion in the *G. lucidum* fruiting body. Subsequent analysis will focus on cell wall composition and signal transduction to understand the mechanism behind the antler-type fruiting bodies of *G. lucidum* under high CO_2_ conditions.

#### 3.2.3. Analysis of Pathways Responding to CO_2_ Concentration

**Figure 6:**
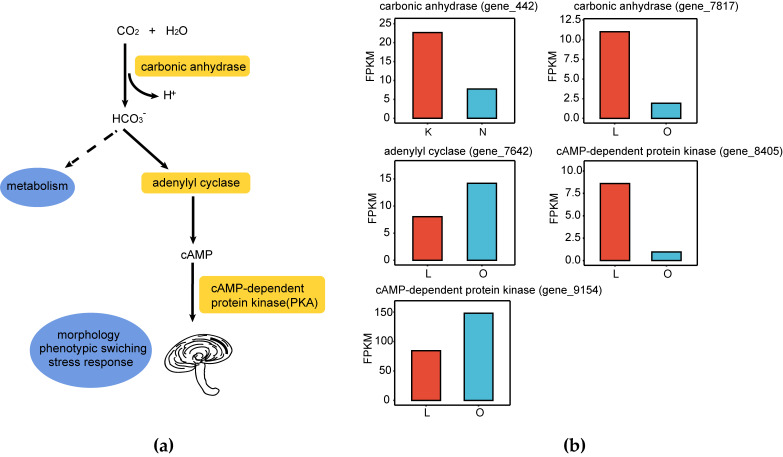
Expression of genes associated with the response to CO_2_ concentration. (**a**) Schematic diagram of CO_2_ signal response and conduction path; (**b**) Bar charts showing the relative expression levels of genes encoding carbonic anhydrase, adenylate cyclase, and cAMP-dependent protein kinase. The *y*-axis represents FPKM values reflecting the levels of gene expression.

Carbonic anhydrase (CA) is a class of zinc metalloenzyme that efficiently catalyzes the reversible reaction of CO_2_ and water to form bicarbonate and protons, thereby regulating the intracellular equilibrium of CO_2_/HCO_3_^−^. This process not only supplies sufficient carbon for cellular metabolism but can also activate adenylate cyclase (AC), subsequently triggering various regulatory mechanisms that influence fungal morphology, communication, and other processes [26,27] (Figure 6a). In this study, four CA family genes were annotated: gene_441, gene_442, gene_7817, and gene_11662. Among these, gene_442 was significantly down-regulated in K vs. N, and gene_7817 was down-regulated in L vs. O (Figure 6b). Liu et al. found that in *F. velutipes* fruiting bodies subjected to high CO_2_ stress for 12 h, the expression levels of *CA-1* and *CA-2* were positively correlated with CO_2_ concentration, while *CA-5* was negatively correlated [28]. Therefore, we speculate that gene_442 and gene_7817 may be key genes involved in the response of the *G. lucidum* fruiting body to elevated CO_2_ concentrations, potentially leading to its morphological changes. In addition, the adenylate cyclase gene (gene_7642), which regulates the synthesis of cAMP, was up-regulated in L vs. O. In addition, differential expression was also observed in the genes encoding cAMP-dependent protein kinase (PKA) located downstream of the cAMP regulatory pathway. Specifically, gene_9154 was significantly up-regulated, whereas gene_8405 was significantly down-regulated in L vs. O.

Heat shock proteins (HSPs) are a group of highly conserved proteins that respond to stress conditions, capable of promoting transcription, translation, protein folding, and the aggregation and degradation of proteins. Here, this study found that high CO_2_ concentrations significantly altered the expression levels of genes encoding HSPs in the fruiting bodies of *G. lucidum*. Specifically, *HSP10* (gene_13499) expression level was significantly up-regulated in J vs. M. Conversely, seven HSP genes were down-regulated in K vs. N, including *HSP20* (gene_11118), *HSP78* (gene_11080, gene_11081, gene_11082, gene_11083), *HSP stil* (gene_402), and Hsp104 (gene_775). In the L vs. O comparison, five HSP genes were significantly down-regulated, including *HSP9/12* (gene_12070, gene_5666), *HSP20* (gene_11118), *HSP90* (gene_11616), and *HSP104* (gene_775). Notably, *HSP20* and *HSP104* were consistently down-regulated in both the K vs. N and L vs. O comparisons.

#### 3.2.4. Analysis of Pathways Related to Cell Division and Proliferation

**Figure 7:**
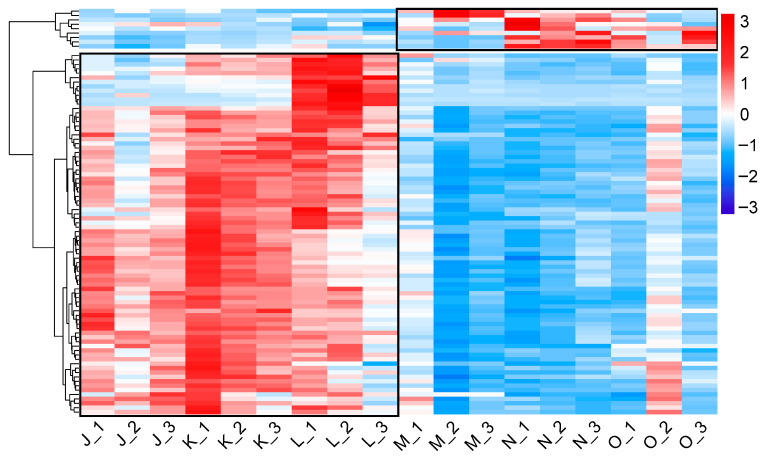
Heatmap of 91 DEGs associated with cell division and proliferation in the comparison group K vs. N. Black lines separate two distinct clades based on expression patterns.

During the second growth stage, the DEGs in the K vs. N comparison were significantly enriched in KEGG pathways related to cell division and proliferation. Specifically, 38 of 42 DEGs in cell cycle-yeast, all 20 DEGs in proteasome, all 22 DEGs in DNA replication, 13 of 14 DEGs in mismatch repair, and 26 of 31 DEGs in meiosis-yeast were down-regulated. A hierarchical clustering heatmap reflected the expression patterns of 91 DEGs annotated to these pathways (Figure 7 and Supplementary Table S5), including DNA mismatch repair protein (msh6, msh-2, MLH1), DNA replication licensing factor (mcm2, mcm3, mcm4, mcm6, mcm7), cell division control protein (CDC6), chromosome segregation protein (sudA), G2/mitotic-specific cyclin (cdc13), conidiation-specific protein 6 (con-6). Collectively, these findings strongly suggest that high CO_2_ concentration inhibits fundamental cell division and proliferation processes. By down-regulating genes essential for cell cycle-yeast, proteasome, DNA replication, mismatch repair, and meiosis-yeast pathways, high CO_2_ concentrations may slow down DNA replication speed, prolong the cell cycle, thereby inhibiting pileus formation. This may lead to solely elongated growth of the fruiting body, ultimately resulting in the formation of slender, antler-shaped *G. lucidum*.

#### 3.2.5. Analysis of Pathways Related to Cell Wall Components

**Figure 8:**
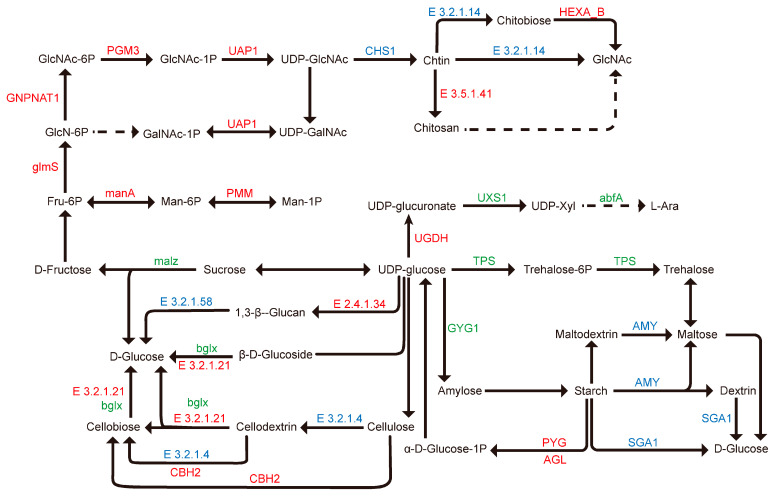
Expression of DEGs enriched in the amino sugar and nucleotide sugar metabolism and starch and sucrose metabolism pathways in the comparison group L vs. O. Genes in red or blue refer to up- or down-regulation responding to CO_2_, respectively. Genes in green refer to both up- and down-regulation in response to CO_2_.

The fungal cell wall is primarily composed of chitin, glucans, and cell wall-associated proteins, which collectively maintain its rigidity, elasticity, tension, and compressive strength [29]. Alterations in cell wall structure and composition are primarily driven by the coordinated action of cell wall-degrading enzymes or proteins, leading to the disruption of wall integrity and a reduction in intercellular connections, ultimately decreasing cellular adhesion. This process involves multiple genes associated with cell wall structure. In the L vs. O comparison group, DEGs were significantly enriched in amino sugar and nucleotide sugar metabolism and starch and sucrose metabolism pathways. From these two pathways, we identified nine differentially expressed cellulose genes (five up-regulated, four down-regulated), five differentially expressed chitin synthase genes (three up-regulated, two down-regulated), 16 differentially expressed chitinase genes (four up-regulated, 12 down-regulated), and one significantly up-regulated chitin deacetylase gene (Figure 8, Supplementary Tables S6 and S7). The differential expression of these cell wall structure-related genes is closely linked to the synthesis of cell wall polysaccharides and energy storage compounds. This suggests that under high CO_2_ conditions, significant changes occur in the biomass and cell wall composition of *G. lucidum* during the third growth stage, indicating an adaptive remodeling of the cell wall.

### 3.3. Integrated Analysis of Transcriptomics and Metabolomics

**Figure 9:**
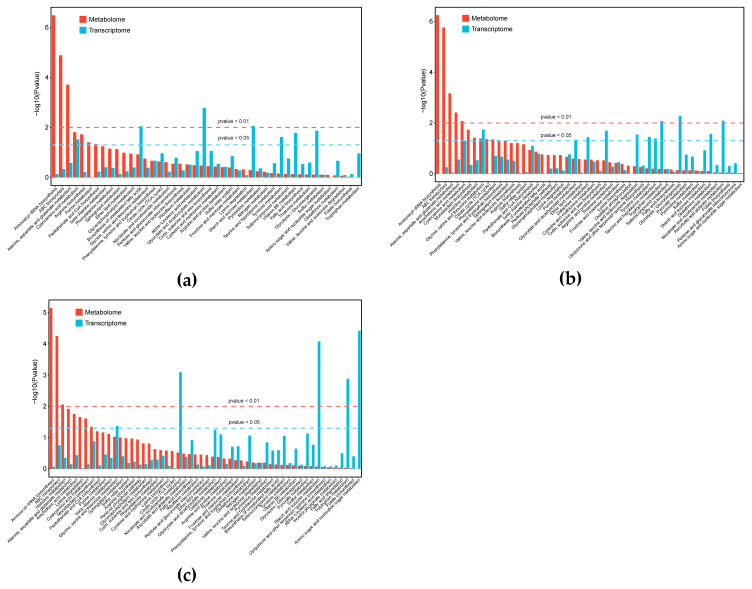

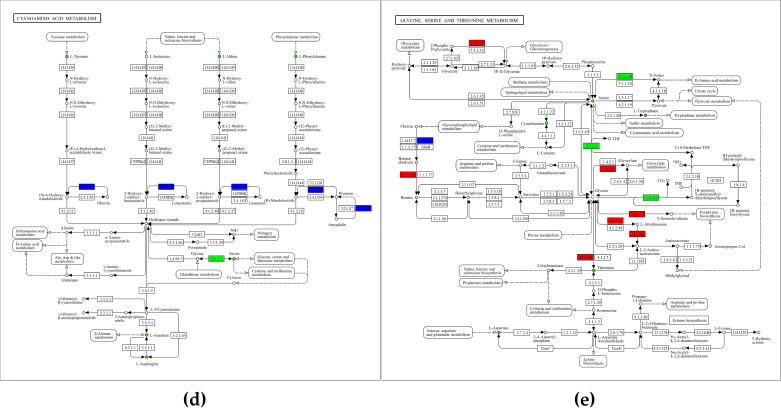
Joint transcriptomic and metabolomic analysis of *G. lucidum*. (**a**–**c**) KEGG enrichment analysis of the DEGs and DAMs in J vs. M (**a**), K vs. N (**b**), and L vs. O (**c**) comparisons. Red and blue bars represent enrichment for DAMs and DEGs, respectively; (**d**) Putative pathway of cyanoamino acid metabolism in *G. lucidum* generated by KEGG analysis; (**e**) Putative pathway of glycine, serine and threonine metabolism in *G. lucidum* generated by KEGG analysis. The boxes highlighted in red or blue represent up- or down-regulation under high CO_2_ concentrations. The boxes highlighted in green mean both up- and down-regulation under high CO_2_ concentrations.

An integrated transcriptomic and metabolomic approach was used to further investigate the response of *G. lucidum* to high CO_2_ concentrations. In the J vs. M, K vs. N, and L vs. O comparisons, KEGG co-enrichment analysis was conducted based on DEGs and DAMs. Specifically, in the J vs. M comparison, both DAMs and DEGs were significantly enriched in cyanoamino acid metabolism (Figure 9a,d). In the K vs. N comparison, both DAMs and DEGs were significantly enriched in glycine, serine and threonine metabolism (Figure 9b,e). However, in the L vs. O comparison, DAMs and DEGs shared no commonly enriched pathways (Figure 9c). Amino acids are essential for fruiting bodies’ growth and development. Beyond their involvement in energy metabolism, they act as osmolytes that alter cellular osmotic pressure. These results indicate that amino acid metabolic pathways in *G. lucidum* are severely affected under high CO_2_ concentrations.

## 4. Discussion

CO_2_ is a key gaseous molecule in the ecosystem and a critical cellular signaling molecule in all organisms. In edible mushroom cultivation, regulation of CO_2_ concentration is primarily achieved by setting sealing and ventilation durations, thereby modulating the morphology of fungal fruiting bodies [13,30,31]. Previous studies have demonstrated that antler-shaped *G. lucidum* fruiting bodies can be produced by controlling ventilation and light conditions [23]. Due to respiration, CO_2_ generated by *G. lucidum* accumulates in the surrounding atmosphere, influencing its physicochemical properties. However, current research remains largely focused on phenotypic observations or simple environmental manipulations. There is still a lack of systematic and in-depth investigation into the complex molecular and metabolic response mechanisms within *G. lucidum* under high CO_2_ stress, particularly regarding its impact on the regulatory synthesis of secondary metabolites and the fruiting body morphogenesis. This study combines transcriptomics and untargeted metabolomics analyses to provide preliminary insights into the physiological changes in *G. lucidum* at the genetic and metabolic levels under elevated CO_2_ cultivation conditions.

### 4.1. Energy Metabolic Remodeling of G. lucidum Under Elevated CO_2_

CO_2_ is closely linked to the synthesis and accumulation of major bioactive compounds in edible fungi. Elevated CO_2_ levels profoundly impact the content of a variety of metabolites, including oxidative stress metabolites, sugars, terpenoids, polyketides, phenolics, and organic acids [23,32,33]. *Ganoderma* triterpenoids are considered the main bioactive components of *G. lucidum*, primarily synthesized via the mevalonate (MVA) pathway [34]. The biosynthesis and accumulation of *Ganoderma* triterpenes are closely related to environmental conditions. Stimuli such as heat stress [35], methyl jasmonate (MeJA) treatment [36], and salicylic acid (SA) treatment [37] can all influence *Ganoderma* triterpene biosynthesis. Furthermore, several signaling pathways, including reactive oxygen species (ROS) [38], Ca^2+^ [39], mitogen-activated protein kinases (MAPK) [40], and nitric oxide (NO) [41], along with their crosstalk, are involved in regulating the synthesis of Ganoderma triterpenoids. Our metabolomic analysis revealed that under high CO_2_ concentration, the accumulation of triterpenoids such as ganoderic acid H, ganoderic acid N, ganoderic acid V, ganoderic acid Mj, ganodermic acid P2, tsugaric acid B, lucidenic acid E2, ganoderiol H was down-regulated, whereas lucidenic acid F, ganoderic acid G, ganoderic acid J, ganoderic acid F, ganolucidic acid D, ganosporeric acid A were significantly up-regulated. Furthermore, the synthesis and accumulation of steroid compounds were also influenced by CO_2_ concentration. In addition to serving as the fundamental components for proteins, peptides, and other nitrogenous biomolecules, amino acids are also integral to key biological processes such as metabolism, survival, interspecies interactions, and virulence. Amino acids function not only as the basic subunits for proteins and peptides but also as key mediators in metabolism, survival, interspecies interactions, and virulence [42]. Our study found that multiple amino acids were generally down-regulated across the J vs. M, K vs. N, and L vs. O comparisons, indicating that the primary metabolic activities of *G. lucidum* were significantly inhibited. These substances are not only precursors for protein synthesis but also important substrates for cellular energy metabolism. Their systematic reduction suggests that *G. lucidum* is facing an energy crisis. It is speculated that under high CO_2_ concentrations, respiration in *G. lucidum* is inhibited, and the cells catabolize amino acids to feed into the tricarboxylic acid (TCA) cycle to rapidly generate ATP for immediate needs.

Heat shock proteins (HSPs) are a highly conserved family of proteins that serve as molecular chaperones in cells. As an integrated network, they participate in the folding of nascent polypeptides, the refolding of metastable proteins, the assembly of protein complexes, the dissociation of protein aggregates, and the degradation of misfolded proteins, which is crucial for maintaining cellular homeostasis [43,44]. Studies have shown that HSPs play a role in responding to various environmental stresses, including pH, temperature, starvation, heavy metals, osmotic stress, and oxidative stress [45,46,47]. Recent research has indicated that HSPs are particularly important in mushroom development, being significantly up-regulated during the fruiting body stage [48,49,50]. However, our transcriptomic analysis revealed that genes encoding HSPs (HSP20, HSP78, HSP90, HSP104) were significantly down-regulated in the K vs. N and L vs. O groups. The induction of HSPs relies on ATP binding and hydrolysis to facilitate their chaperone function. Therefore, the down-regulation of HSPs in the *G. lucidum* fruiting body under high CO_2_ may represent an adaptive strategy. This suggests that, when facing energy scarcity, the cells actively curtail the energy-intensive process of protein quality surveillance, prioritizing the limited ATP for maintaining ionic homeostasis and fundamental metabolism.

### 4.2. High CO_2_ Concentrations Inhibit Cell Division and Proliferation Pathways in G. lucidum

Morphological changes in fruiting bodies are an adaptive strategy of edible fungi in response to elevated CO_2_. Studies have shown that elevated CO_2_ can affect hyphae differentiation, cap expansion, and stipe elongation [13,18]. In the phenotypic observation of the *G. lucidum* high CO_2_ response, the most striking phenomenon is the formation of antler-shaped malformed fruiting bodies. However, the mechanism of morphogenesis for these antler-shaped fruiting bodies under elevated CO_2_ is poorly understood, with related research primarily focusing on the CO_2_-induced morphological changes in *F*. *velutipes* and *P*. *ostreatus*. Previous omics studies found that high CO_2_ concentrations adversely affect gene expression of the ubiquitin-proteasome system and cell cycle-yeast pathway, and can specifically repress PHO80-like cyclin genes, thereby inhibiting pileus development in *F. velutipes* [12,19]. Similar findings were observed in our study. Transcriptomic analysis showed that in K vs. N, the vast majority of DEGs in pathways such as cell cycle-yeast, proteasome, DNA replication, mismatch repair, meiosis-yeast, and MAPK signaling pathway-yeast were down-regulated. This indicates that the inhibition of cell division and proliferation in *G. lucidum* by elevated CO_2_ is particularly significant during the second growth stage (K vs. N), where the DNA replication rate and cell cycle progression were severely delayed. A previous proteomic study reported that the sexual differentiation process protein Isp4 was inhibited in *P. ostreatus* under high CO_2_ concentrations [18]. In this study, we annotated six Isp4 genes using the Swiss-Prot database. In J vs. M, two genes (gene_11404, gene_11401) were significantly up-regulated, whereas in K vs. N and L vs. O, two Isp4 genes were significantly down-regulated in each comparison (gene_7059 and gene_8047; gene_11404 and gene_8047, respectively). This suggests that under high CO_2_ concentrations, the sexual differentiation process of *G. lucidum* may be inhibited as the fruiting body matures.

DNA replication is crucial for premeiotic replication, nuclear movement, and meiosis during mushroom maturation [51,52]. The proteasome participates in many fundamental cellular functions, such as cell cycle regulation, cell differentiation, signal transduction pathways, stress signaling, and apoptosis, and can degrade unnecessary proteins via the ubiquitin-proteasome pathway [53,54]. Hossain et al. reported that the proteasome controls fungal morphogenesis via functional connections with Hsp90 and cAMP-protein kinase A signaling [55]. The MAPK pathway is a vital signal transduction pathway involved in regulating various physiological activities, including cell function (proliferation, gene expression, differentiation, mitosis, survival, and apoptosis), maintenance of cell wall integrity, fruiting body development, stress responses, and conidiation [56,57,58]. Carbonic anhydrase (CA) is a metalloenzyme that catalyzes the interconversion of CO_2_ and bicarbonate. In Saccharomyces cerevisiae, CA family proteins have been reported to participate in regulatory mechanisms such as CO_2_ sensing and pseudohyphal development [59]. This regulatory mechanism involves CA activating adenylate cyclase, leading to elevated cAMP levels, which activates the cAMP/PKA pathway to control various cellular processes [27]. Our study found that the expression levels of carbonic anhydrase genes (gene_442, gene_7817), the adenylate cyclase gene (gene_7642), and cAMP-dependent protein kinase (PKA) genes (gene_9154, gene_8405) changed significantly under elevated CO_2_. Previous studies have suggested that MAPK and cAMP/PKA signaling pathways may be involved in regulating sexual development in fungi [60,61]. Therefore, we hypothesize that CO_2_ may perceive and transduce stress signals through these related pathways, inhibiting the expression of key genes for cell division and proliferation, thereby blocking the normal differentiation and development of the pileus. This inhibition works in concert with the reconstruction of energy metabolism: the energy shortage prevents cells from supporting the highly energy-intensive division process, while the cessation of cell division further exacerbates the overall decline in metabolic activity. Ultimately, the *G. lucidum* fruiting body is unable to form a normal pileus and can only undergo stipe elongation, resulting in the typical antler-shaped malformation. This finding not only reveals the molecular mechanism of *G. lucidum* morphogenesis under CO_2_ stress but also provides a new perspective on how fungi adapt to environmental pressure by inhibiting proliferation.

### 4.3. High CO_2_ Concentrations Regulate Cell Wall Synthesis and Remodeling in G. lucidum

The fungal cell wall is a highly dynamic structure that undergoes significant rearrangement in response to environmental conditions or imposed stresses [62]. Complex signaling networks enable the cell wall integrity (CWI) pathway to be activated by various types of stress and provide the response necessary to maintain fungal cell viability [63]. Many reports have elucidated the importance of the fungal cell wall under abiotic stress [64,65,66]. In *P. ostreatus*, proteins associated with hydrolase activity, including several amidohydrolases and cell wall synthesis proteins, exhibited high levels of expression under high CO_2_ concentrations [18]. Sietsma’s research found that CO_2_ affects the synthesis of R-glucan, indirectly influencing the pileus growth of *S*. *commune* by altering cell wall composition [67].

In the transcriptomic analysis of this study, GO and KEGG enrichment analyses indicated that in the third growth stage of *G. lucidum* (L vs. O), DEGs were primarily involved in biological processes and molecular functions such as the carbohydrate metabolic process, hydrolase activity, carbohydrate binding, and structural constituent of the cell wall. The amino sugar and nucleotide sugar metabolism and starch and sucrose metabolism pathways were significantly enriched. The DEGs included hydrophobins (HYD1, SC1, SC3, SC4), members of chitin- and glucan-active glycoside hydrolases (GH) and glycosyl transferase (GT) families, and chitin deacetylases.

Among these, hydrophobins are widely present in fungal cell walls, imparting a hydrophobic nature to the cell wall. The gene Fv-hyd1 encoding the hydrophobin is specifically expressed during fruiting body development in *F. velutipes* [68]. Silencing the hydrophobin hyd1 gene decreases the resistance of *G. lucidum* mycelia to heat, cell wall, and salt stress [69]. In *S. commune*, the genes *SC1* and *SC4* are only active in the dikaryon and the encoded hydrophobins accumulate in hyphal walls of developing fruit bodies. SC3 mediates attachment of hyphae to hydrophobic surfaces and the encoded hydrophobins accumulate in walls of individually growing aerial hyphae, while SC4 connects gas channels within fungi fruiting bodies, potentially preventing collapse of air channels allowing continuous gas exchange [70,71,72]. Interestingly, this study found that in K vs. N, *SC1* (gene_1850) and *SC4* (gene_10364, gene_10358, gene_10359) were significantly up-regulated, and in L vs. O, *SC1* (gene_1850) and *SC4* (gene_10364) were also significantly up-regulated. These genes may be involved in gas exchange within the hyphal walls of the *G. lucidum* fruiting body under high CO_2_. Furthermore, cell wall remodeling is primarily facilitated by endogenous carbohydrate-active enzymes (CAZymes), whose expression is generally regulated by specific genes. In L vs. O, the expression of genes encoding chitinase (GH18), chitin synthase (GT2), cellulase (GH5), and alpha-glucosidase (GH31) changed significantly. It is thus speculated that changes in CAZyme gene expression may contribute to the adaptive remodeling of the *G. lucidum* fruiting body cell wall under elevated CO_2_.

In the fruiting body, the cell wall not only bears structural and defensive functions but must also be sufficiently plastic to support changes in shape and size during development [73]. Compared to normal fruiting bodies, the cell walls in *L. edodes* fruiting bodies with abnormal pilei are thicker and cell wall anabolism is active, enhancing the adaptability [74]. Stipe growth may be achieved by hydrolytic enzymes breaking chitin-glucan cross-links [75]. Therefore, we speculate that the adaptive remodeling of the cell wall under high CO_2_ concentrations not only enhances the resistance of *G. lucidum* to osmotic stress but also directly influences fruiting body morphogenesis. In addition, localized thickening and non-uniform elongation of the cell wall may be the structural basis for the excessive stipe elongation and hindered pileus development.

## 5. Conclusions

This study conducted the transcriptome and metabolome analysis to uncover the molecular response of medicinal fungi *G. lucidum* under high CO_2_ concentrations. We demonstrate that elevated CO_2_ triggers a profound reprogramming of energy metabolism, significantly altering the accumulation of key metabolites like triterpenoids, steroids, and amino acids. Critically, during a key developmental window, high CO_2_ suppresses genes governing the cell cycle, DNA replication, and proteasome function. This systemic inhibition of cell proliferation is the likely direct molecular cause of the failure to form a normal pileus, resulting in the characteristic “antler-like” phenotype. In later stress stages, the fungus actively remodels its cell wall by modulating genes for carbohydrate-active enzymes and hydrophobins, a strategy we hypothesize enhances osmotic stress resistance and influences morphogenesis by altering cell wall mechanics. In summary, our findings indicate that the inhibition of cell division, energy metabolism reprogramming, and cell wall remodeling act in concert to shape the unique, stress-adaptive phenotype of *G. lucidum*. Our work provides important insights into the underlying mechanism by which high CO_2_ concentrations regulate the development of the *G. lucidum* fruiting body, which would facilitate its high-quality and efficient cultivation.

## Data Availability

The RNA-seq data in this study have been deposited in the NCBI repository (https://www.ncbi.nlm.nih.gov/ (accessed on 11 November 2025)), accession number PRJNA1358748.

## Supplementary Materials

The following supporting information can be downloaded at: https://www.mdpi.com/article/10.3390/jof12010005/s1, Figure S1: Venn diagram of DAMs; Figure S2: Pearson correlation analysis between all transcriptome samples; Table S1: Metabolite annotation results; Table S2: The differentially accumulated metabolites in J vs. K, K vs. L, J vs. L, M vs. N, N vs. O, M vs. O, J vs. M, K vs. N, L vs. O; Table S3: Statistics of the quality and comparisons with the reference genome; Table S4. The differentially expressed genes in J vs. K, K vs. L, J vs. L, M vs. N, N vs. O, M vs. O, J vs. M, K vs. N, L vs. O; Table S5: 91 DEGs associated with cell division and proliferation in K vs. N; Table S6: 34 DEGs enriched in amino sugar and nucleotide sugar metabolism (ko00520) in L vs. O; Table S7: 28 DEGs enriched in starch and sucrose metabolism (ko00500) in L vs. O.
