# Supplementary material for: Combined Transcriptomic and Metabolomic Analyses of the Response of Ganoderma lucidum to Elevated CO2
Source: J Fungi (Basel). 2025 Dec 20;12(1):5. doi: 10.3390/jof12010005 (PMC12842923; doi:10.3390/jof12010005)
Supplement: Supplementary file 1 [file jof-12-00005-s001.zip › Supplementary figures.pdf]

Supplementary figures:

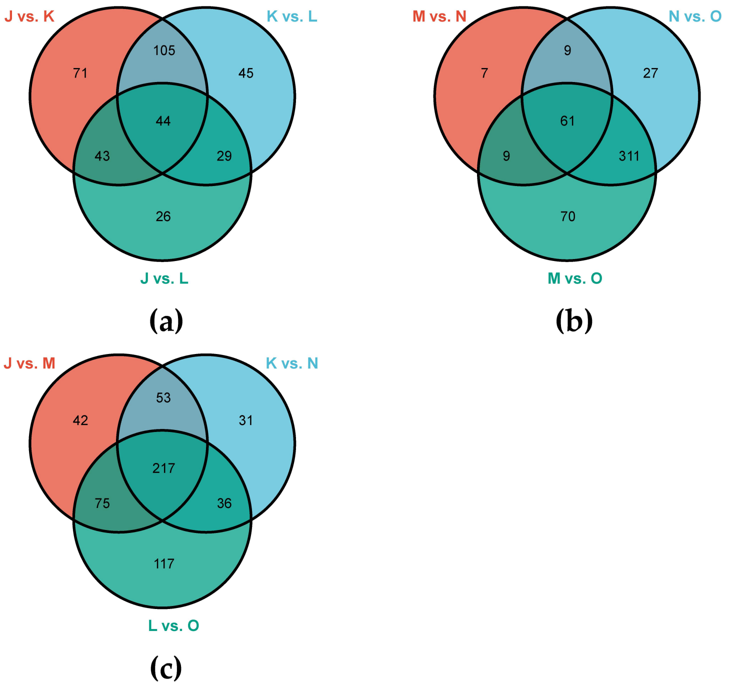

Figure S1. Venn diagram of DAMs. (a)Venn diagram of DAMs in J vs. K, K vs. L and J vs. L; (b) Venn diagram of DAMs in M vs. N, N vs. O and M vs. O; (c) Venn diagram of DAMs in J vs. M, K vs. N and L vs. O

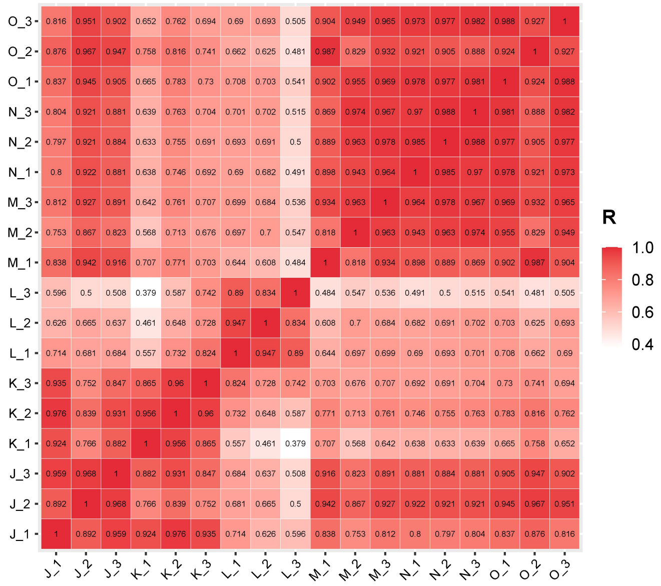

Figure S2. Pearson correlation analysis between all transcriptome samples.
